# Nanoimprinted TiO_2_ Metasurfaces with Reduced Meta-Atom Aspect Ratio and Enhanced Performance for Holographic Imaging

**DOI:** 10.3390/ma17102273

**Published:** 2024-05-11

**Authors:** Kaiyu Zhang, Yuqi Lin, Yang Qiu, Xingyan Zhao, Shaonan Zheng, Yuan Dong, Qize Zhong, Ting Hu

**Affiliations:** 1 School of Microelectronics, Shanghai University, Shanghai 201899, China; 2 Shanghai Key Laboratory of Intelligent Connected Vehicle Interaction Chip and System, Shanghai University, Shanghai 200444, China

**Keywords:** nanoimprint, metasurface, hologram, titanium oxide, aspect ratio

## Abstract

Metasurface holograms, with the capability to manipulate spatial light amplitudes and phases, are considered next-generation solutions for holographic imaging. However, conventional fabrication approaches for meta-atoms are heavily dependent on electron-beam lithography (EBL), a technique known for its expensive and time-consuming nature. In this paper, a polarization-insensitive metasurface hologram is proposed using a cost-effective and rapid nanoimprinting method with titanium dioxide (TiO_2_) nanoparticle loaded polymer (NLP). Based on a simulation, it has been found that, despite a reduction in the aspect ratio of meta-atoms of nearly 20%, which is beneficial to silicon master etching, NLP filling, and the mold release processes, imaging efficiency can go up to 54% at wavelength of 532 nm. In addition, it demonstrates acceptable imaging quality at wavelengths of 473 and 671 nm. Moreover, the influence of fabrication errors and nanoimprinting material degradation in terms of residual layer thickness, meta-atom loss or fracture, thermal-induced dimensional variation, non-uniform distribution of TiO_2_ particles, etc., on the performance is investigated. The simulation results indicate that the proposed device exhibits a high tolerance to these defects, proving its applicability and robustness in practice.

## 1. Introduction

Metasurfaces, composed of subwavelength antenna arrays, possess unique optical responses [1,2,3] and remarkable optical modulation capabilities, enabling the realization of novel optical effects and attractive functions not observed in nature. Therefore, they have been applied in various fields, such as metalens [4,5], holographic devices [6,7,8], optical cloaks [9], and color filters [10,11]. Metasurface holography allows for arbitrary complex wavefront shaping within a rather compact footprint, overcoming limitations associated with traditional holography techniques, such as low resolution, restricted field of view (FOV), and multi-order-diffraction-induced noises [3]. Consequently, it has been regarded as one of the most promising technologies for holographic imaging. However, considering the dimension of meta-atoms is typically in the range of 100 to 400 nanometers [12,13,14], the fabrication of metasurfaces usually relies on electron-beam lithography (EBL) which is costly and time-demanding. Worse, the aspect ratio of meta-atoms must be high enough to ensure acceptable imaging quality, resulting in reduced overall yield and increased process difficulty.

Compared with EBL, nanoimprint lithography (NIL) offers significant advantages including lower cost, higher throughput [5,15], and equal resolution. In the NIL process, desired masks or devices can be produced in seconds or minutes using a mold, eliminating the need for prolonged electron beam exposure [16]. While the fabrication of the silicon (Si) master still requires EBL, the negative molds derived from it can be reused, enabling large-scale and rapid manufacturing subsequently, therefore reducing costs and uplifting the throughput [17,18,19]. NIL can be divided into two main categories. The first one involves imprinting the photoresist layer with the negative mold to obtain depositing or etching windows repeatedly, substituting the lengthy and costly EBL process. The second one is to imprint fluid materials (usually polymer-based) through the negative mold to create the same device as the Si master directly.

For metasurface holograms operating in the visible light spectrum, the meta-atom material necessitates a high refractive index (*n*) to achieve effective light manipulating capabilities. A popular approach is to grow a pure titanium dioxide (TiO_2_) film using atomic layer deposition (ALD) [20], followed by electron-beam lithography (EBL) patterning of photoresist and dry etching. But with polymer-based materials, such as mixtures of TiO_2_ nanoparticles and base epoxies, thanks to their sufficient fluidity, the fabrication of metasurface can be achieved in merely one step of molding with no depositing, patterning, or etching procedures. This means the costly ALD and EBL equipment can be avoided. Nevertheless, due to the high aspect ratio of meta-atoms, defects including incomplete filling of holes, fracture or even missing pillars, sloped sidewalls, etc., arise during negative mold fabrication and the following replication processes, which ultimately leads to incomplete matching with the Si master.

In this work, we propose a novel polarization-insensitive metasurface hologram based on NIL of the TiO_2_ nanoparticle loaded polymer (NLP). Compared to prior research, in which the aspect ratio of nanoimprinted meta-atoms typically exceeds 6, resulting in an imaging efficiency of about 48% at a wavelength of 532 nm [21], we have successfully reduced the aspect ratio to below 5. This reduction proves advantageous for several critical processes, including Si master etching, nanoimprinting of TiO_2_ NLP, and final demolding. Meanwhile, the imaging efficiency can be raised to nearly 54%. In addition, we extensively explore the impact of key fabrication and material defects such as residual layer thickness, meta-atom fracture or missing meta-atoms, curing-induced dimensional change, uneven mixing of TiO_2_ particles, etc., on the optical performance. The simulation outcomes suggest that our device exhibits a high tolerance to such defects, enhancing its practical applicability and robustness.

## 2. Materials and Methods

### 2.1. TiO_2_ NLP and Molding Materials

From the perspective of the refractive index and extinction coefficient, pure TiO_2_ might be the preferred choice for metasurface devices working within the visible spectrum. However, the corresponding EBL, ALD, and dry etching are of high expense, low throughput, and long processing time; therefore, we selected TiO_2_ NLP (Pixelligent, Baltimore, MD, USA) as the metasurface material. It has a relatively low viscosity of 3.6 cP at room temperature (23 °C), which is advantageous for the filling of small holes. At a wavelength (λ) of 532 nm, the refractive index and transmittance are approximately 1.975 and 0.92. Its basic information is summarized in Table 1.

As the small features on the negative mold will experience elongation and distortion in every replication, it is necessary for them to have a relatively high Young’s modulus to avoid deformation or fracture. However, to ease the demolding process, the negative mold is supposed to be flexible enough. So, to reach a balance between long mold lifetime and demolding smoothness, hard polydimethylsiloxane (h-PSDMS) with a Young’s modulus of about 9.8 MPa (Gelest, Inc., Morrisville, PA, USA) and regular PDMS with a Young’s modulus of about 1.7 MPa (Merck, Shanghai, China) are combined.

### 2.2. Design of Metasurface Hologram

**Figure 1:**
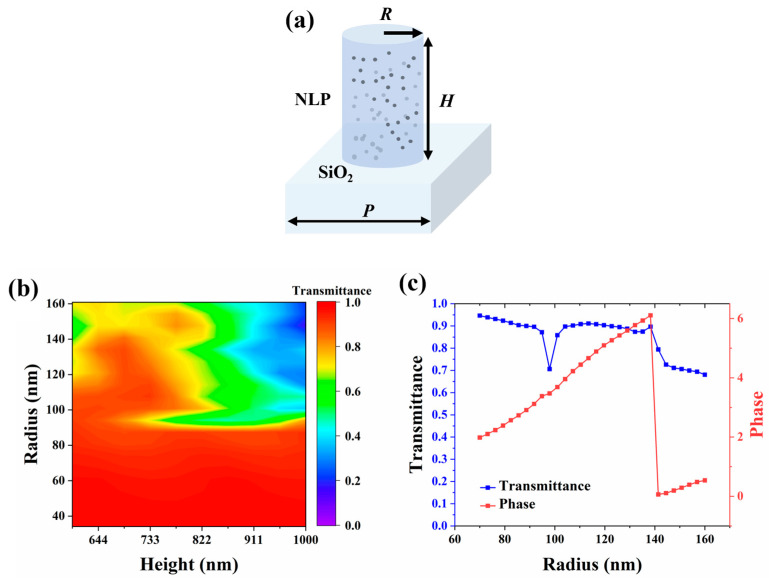
(**a**) Schematic of a single TiO_2_ NLP meta-atom based on the TiO_2_ NLP on the glass substrate. The height, radius, and period are noted as *H*, *R*, and *P,* respectively. (**b**) Transmittance of the meta-atoms at λ = 532 nm with a fixed period of 420 nm. (**c**) Transmittance and phase of the meta-atoms at λ = 532 nm with a period of 420 nm, height of 680 nm, and radii from 70 to 160 nm.

To facilitate the demolding process, all meta-atoms are designed as cylinders on a glass substrate with an *n* value of 1.45 (Figure 1a). As the period (*P*) increases, the multi-order-diffraction becomes more profound, leading to a reduction in the field of view (FOV). Therefore, we fixed the period at 420 nm and varied the radius (*R*) and height (*H*) of the cylinders to generate the transmittance spectrum, as shown in Figure 1b. The scanning ranges are from 600 to 1000 nm and from 40 to 160 nm, respectively. The finalized structural parameters are as follows: the height is 680 nm, and the radii are from 70 to 160 nm. Hence, the highest aspect ratio of the cylinders is around 4.85. The transmittance and phase spectrum are obtained by scanning the radii from 70 to 160 nm, as shown in Figure 1c.

**Figure 2:**
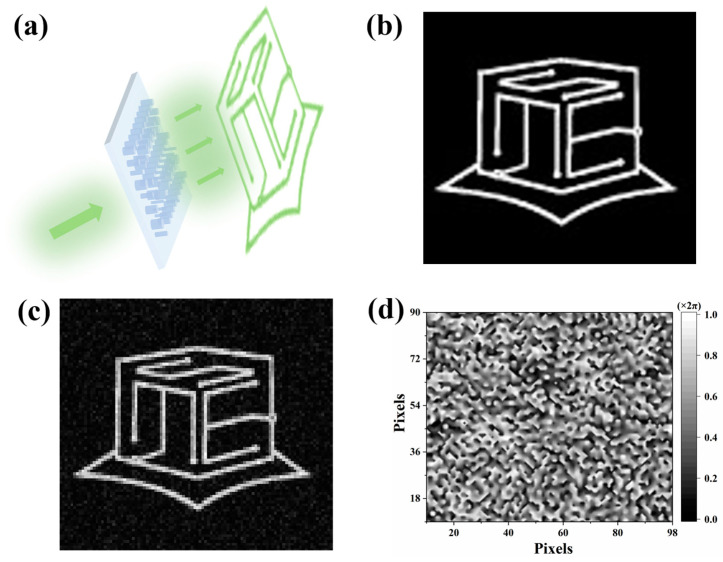
(**a**) Schematic of how the metasurface hologram works. (**b**) Target image. (**c**) Holographic image created by the Gerchberg–Saxton (GS) algorithm. (**d**) Phase information of the metasurface hologram.

As demonstrated in Figure 2a, when incident light interacts with a metasurface that carries the phase information of a target image, the resulting far-field pattern will display the target image. To extract the necessary phase information required for this process, we adopt the Gerchberg–Saxton (GS) algorithm [22], which employs an iterative approach to minimize the discrepancy between the target and holographic images. More specifically, this algorithm first extracts the original amplitude (or intensity) of the target image which is shown in Figure 2b, then attaches a random phase to it. After that, through inverse fast Fourier transformation (IFFT), the complex amplitude of the object surface (i.e., metasurface) can be obtained. Eventually, through amplitude normalization, phase extraction, and fast Fourier transformation (FFT), the holographic image (i.e., far field) is obtained, as shown in Figure 2c. To guarantee high-quality reconstruction, the discrepancy between the target and holographic images should be compared to determine the parameters for the next iteration. As long as a significant discrepancy (more than 0.005) is found, the integration of the original amplitude and the extracted phase will be processed. This process is followed by another iteration utilizing the IFFT method, initiating the next iterative step. Ultimately, the extracted phase information reflects the phase distribution of the metasurface hologram, as shown in Figure 2d. The model of the metasurface hologram is obtained by configuring meta-atoms of varying sizes according to the phase distribution. The target image has a resolution of 90 by 98 pixels, resulting in a total of 90 by 98 meta-atoms.

### 2.3. Nanoimprinting of Metasurface Hologram

To fabricate the master, a layer of photoresist is spin-coated, then patterned using EBL on a Si substrate as the etching mask, followed by deep reactive ion etching (DRIE) to form the desired pillars. After etching, the master undergoes a fluorine-based material surface treatment to facilitate the subsequent demolding process. Then, a layer of the h-PDMS is coated on the treated master and cured in a vacuum environment at 60 °C for 60 min to create a reverse replica of the desired metasurface patterns. This step is repeated with the regular PDMS to roughly adjust the final thickness and flexibility of the mold, which is critical to the imprinting and demolding processes. After the curing of PDMS, the master can be detached, and the surface of the mold should be fluorinated to further improve the demolding properties.

**Figure 3:**
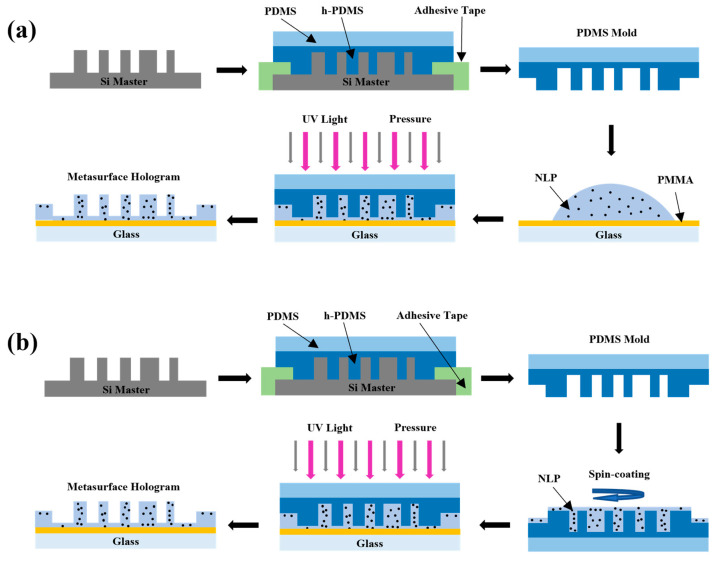
(**a**) Schematic of a typical nanoimprinting process in fabricating the metasurface hologram. The TiO_2_ NLP is directly poured onto the glass substrate, then compressed by the mold. (**b**) Schematic of the optimal nanoimprinting process in fabricating the metasurface hologram. The TiO_2_ NLP is spin-coated on the mold to control the residual layer thickness, then compressed to the glass substrate.

The typical imprinting method involves directly pouring a sufficient amount of the TiO_2_ NLP on a precleaned glass substrate to cover the desired area, then compressing the mold against it to ensure good contact, as depicted in Figure 3a. Nevertheless, the downside to this is that the thickness of the residual layer at the mold–substrate interface cannot be precisely controlled, ultimately influencing the imaging quality and efficiency. Therefore, we propose an optimal implementation, which is to spin-coat the TiO_2_ NLP onto the mold, so that the thickness of the residual layer can be adjusted by the spinning configuration, as shown in Figure 3b. In either case, before the molding process, a thin layer of adhesion promotor made of polymethylmethacrylate (PMMA) is required between the glass substrate and the TiO_2_ NLP. In addition, ultraviolet light and heat are needed to accelerate the curing process during compression.

## 3. Results and Discussion

### 3.1. Imaging Quality

**Figure 4:**
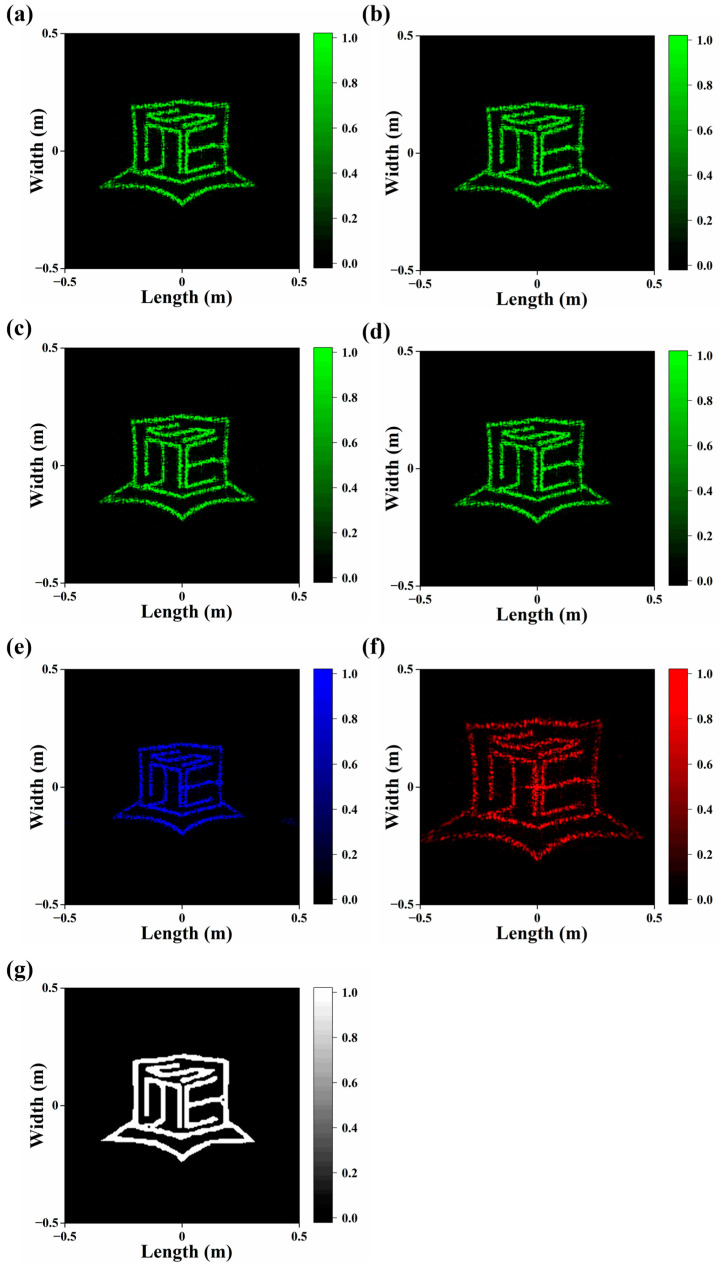
Far-field images for different incident light. (**a**) *x*-linear polarized light with wavelength of 532 nm. (**b**) *y*-linear polarized light with wavelength of 532 nm. (**c**) Right-hand circularly polarized light with wavelength of 532 nm. (**d**) Right-hand circularly polarized light with wavelength of 532 nm. (**e**) *x*-linear polarized light with wavelength of 473 nm. (**f**) *x*-linear polarized light with wavelength of 671 nm. (**g**) Target image region used for imaging efficiency calculation.

The optical performance of the proposed metasurface hologram is evaluated using the finite-difference time-domain (FDTD) method. First, as *x*-linear polarized light at wavelength of 532 nm hits the metasurface hologram, the far-field electric field distribution information is extracted to receive the resulting image, as shown in Figure 4a. After that, *y*-linear polarized, right-hand circularly polarized (RCP), and left-hand circularly polarized (LCP) light of the same wavelengths are employed; the corresponding results are given by Figure 4b–d, respectively. It can be seen that the imaging quality remains acceptable regardless of the polarization state at a wavelength of 532 nm. When incident light at wavelengths of 473 nm and 671 nm pass through the metasurface hologram, the resulting images are also displayed clearly (Figure 4e,f). However, it is observed that the size of the resulting images changes with the variation in wavelength.

### 3.2. Imaging Efficiency

The imaging efficiency (*eff*) can be defined by the following equation, where *T* is the transmittance of the metasurface and *W*_1_ and *W*_2_ represent the optical powers of the target image region and the far-field, respectively [21,23].

*eff = T* × (*W*_1_/*W*_2_). (1)

The target image region is extracted from the far-field patterns and manually patched, as demonstrated by Figure 4g. For the *x*-linear polarized light at a wavelength of 532 nm, the imaging efficiency is about 54%. It should be noted that the imaging efficiency is influenced by various factors, especially the pattern and the resolution of the target image.

### 3.3. Effect of Fabrication Errors

It is necessary to investigate fabrication errors in terms of meta-atom fracture or loss, base epoxy shrinkage, residual layer, distributional non-uniformity of loaded particles, etc., as all of them may influence the imaging performance of the metasurface hologram.

**Figure 5:**
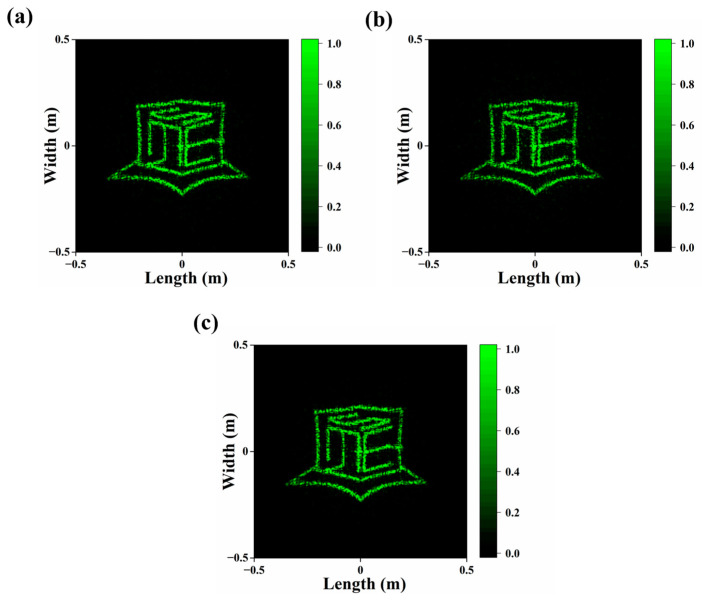
Far-field images for different fabrication errors. (**a**) Height of cylinders with diameters less than 160 nm randomly decreased by 480 nm or more. (**b**) Height of cylinders with diameters less than 200 nm randomly decreased. (**c**) Bottom diameter of all cylinders increased by 60 nm.

For meta-atom fracture or loss, because structures with higher aspect ratios are more vulnerable to incomplete filling during imprinting and distortion during demolding, two cases are analyzed. In the first case, meta-atoms with diameters smaller than 160 nm are assumed to experience a random height decrease by 480 nm or more. The calculated transmittance and imaging efficiency are 78.1% and 50.9%. In the second case, more meta-atoms are assumed to suffer since the upper limit of the diameter expands to 200 nm; the height decrease is from 0 to 680 nm, which is more random. The calculated transmittance and imaging efficiency are 76.4% and 46.2%. In both cases, the far-field imaging patterns are still acceptable (Figure 5a,b).

**Figure 6:**
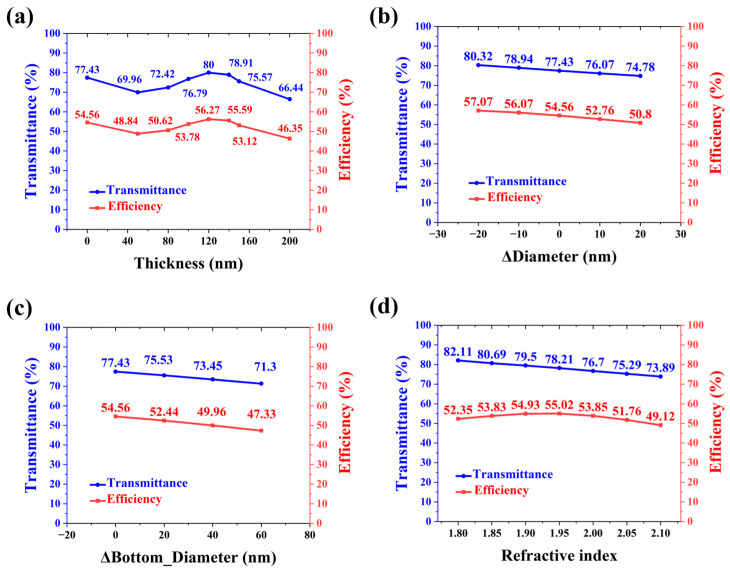
Transmittance and imaging efficiency for different fabrication errors. (**a**) Residual layer thickness increased from 0 to 200 nm. (**b**) Shrinkage due to solution evaporation and swell caused by moisture absorption. The height is fixed; the variation of diameter is from −20 to 20 nm. (**c**) Sloped sidewall caused by inappropriate etching. The top diameter is fixed, the variation of the bottom diameter is from 0 to 60 nm. (**d**) Refractive index from 1.8 to 2.1.

The residual layer can usually be eliminated by an extra maskless DRIE process after curing, but the downside is a slight height decrease in the meta-atoms. In addition, the existence of residual polymer links the meta-atoms, improving their overall adhesion to the glass substrate. Interestingly, it has a mixed influence on the optical performance of the metasurface hologram. When the thickness grows from 0 to 200 nm, both transmittance and imaging efficiency witness fluctuations, and the maximums (56.27% and 80%) occur at thicknesses of 120 nm, as shown in Figure 6a.

On one hand, to enhance the fluidity of the NLP, sometimes evaporable solution is deliberately added to the mixture, resulting in shrinkage after curing. On the other hand, polymer-based structures may swell after absorption of moisture during usage. Therefore, to take these two situations into consideration, the transmittance and imaging efficiency are measured after the diameter of all meta-atoms is increased and decreased by 10 and 20 nm. From Figure 6b, it can be concluded that both transmittance and imaging efficiency grow as the diameter decreases. 

As straight sidewalls are not easy to form while dry-etching the Si master, sometimes a via with a sloped sidewall appears, which leads to the cylinder having a sloped sidewall profile after the replication. To figure out how the sidewall profile can impact the optical performance, the lower diameter of all cylinders is increased by 20, 40, and 60 nm while keeping the upper diameter unchanged. The sloped angles are 0.84°, 1.68°, and 2.52°. The results reveal that the transmittance and imaging efficiency see a downward trend of nearly 7% with the diameter increase. However, it is worth noting that the far-field imaging pattern remains acceptable despite these shape changes, as given by Figure 5c.

**Figure 7:**
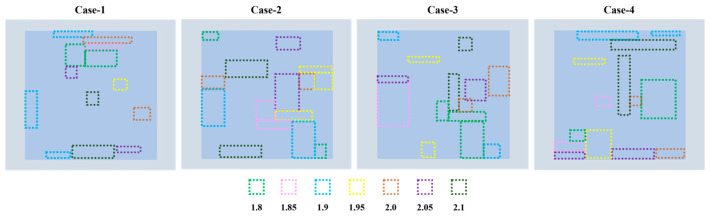
Different cases for transmittance and imaging efficiency calculation. In each case, a random number of regions with various sizes and locations are selected from the metasurface and assigned various refractive indices ranging from 1.8 to 2.1.

A common issue with NLP is the non-uniform distribution of the loaded particles due to insufficient mixing or precipitation. Consequently, the refractive index in practice might be different from the vendor-specified one; thus, the transmittance and imaging efficiency with different refractive indices are calculated. As shown in Figure 6d, the results suggest that the transmittance decreases continuously from 82.11% to 73.89% when the refractive index grows from 1.8 to 2.1. Nevertheless, the imaging efficiency sees a different trend. It reaches a maximum of 55.02% as the refractive index is about 1.95 and drops to a minimum of roughly 49% when the refractive index is 2.1. Another scenario might be that the non-uniform distribution of the loaded particles impacts the nonlinear characteristics of the meta-atom material, so the refractive indices across the metasurface are not the same [24]. Therefore, four cases are analyzed to investigate this scenario. In each case, a random number of regions with different sizes on the metasurface are selected, in which the meta-atoms are assigned various refractive indices from 1.8 to 2.1, as demonstrated in Figure 7. The result, as shown in Table 2, indicates that the non-uniformity has limited influence on the optical performance, because neither the transmittance nor the imaging efficiency exhibits significant changes.

## 4. Conclusions

In this work, we report a cost-efficient polarization-insensitive metasurface hologram at a wavelength of 532 nm through nanoimprinting of the TiO_2_ NLP, which demonstrates enhanced transmittance and imaging efficiency of about 77% and 54%, with acceptable imaging quality at wavelengths of 473, 532 and 671 nm. The meta-atoms have a low aspect ratio of around 4.86, which is advantageous for the molding and demolding processes. Moreover, the effects of commonly seen errors in fabrication and material on the optical performance are explored. When the high aspect ratio cylinders experience a height decrease of 480 nm or more, the imaging efficiency goes down to 50.9%. Due to shrinkage of the base epoxy, both transmittance and imaging efficiency witness an upward trend of nearly 6%. Apart from that, the distributional non-uniformity of loaded particles has no dramatic effect on the optical performance.

## Figures and Tables

**Table 1:** Basic information on TiO_2_ NLP.

Name	Appearance	Viscosity	Refractive Index	Transmittance	Young’s Modulus	Hardness
PixNIL ST6	Off-white	3.6 cP @ 23 °C	~1.975 @ λ = 532 nm	~0.92 @ λ = 532 nm	11.6 GPa	0.64 GPa

**Table 2:** Transmittance and imaging efficiency for 4 cases.

Case	1	2	3	4
Transmittance	79.16%	79.13%	79.15%	79.12%
Imaging Efficiency	55.25%	55.23%	55.24%	55.23%

## Data Availability

Data are available upon request.
